# A systematic study of traditional Chinese medicine treating hepatitis B virus-related hepatocellular carcinoma based on target-driven reverse network pharmacology

**DOI:** 10.3389/fcimb.2022.964469

**Published:** 2022-08-15

**Authors:** Xiaofeng Yin, Jinchuan Li, Zheng Hao, Rui Ding, Yanan Qiao

**Affiliations:** ^1^ Department of Neurosurgery, Second Hospital of Shanxi Medical University, Taiyuan, China; ^2^ Department of Pharmacy, Second Hospital of Shanxi Medical University, Taiyuan, China

**Keywords:** traditional Chinese medicine, hepatitis B virus-related hepatocellular carcinoma, target-driven, reverse network pharmacology, a systematic study

## Abstract

Hepatocellular carcinoma (HCC) is a serious global health problem, and hepatitis B virus (HBV) infection remains the leading cause of HCC. It is standard care to administer antiviral treatment for HBV-related HCC patients with concurrent anti-cancer therapy. However, a drug with repressive effects on both HBV infection and HCC has not been discovered yet. In addition, drug resistance and side effects have made existing therapeutic regimens suboptimal. Traditional Chinese medicine (TCM) has multi-ingredient and multi-target advantages in dealing with multifactorial HBV infection and HCC. TCM has long been served as a valuable source and inspiration for discovering new drugs. In present study, a target-driven reverse network pharmacology was applied for the first time to systematically study the therapeutic potential of TCM in treating HBV-related HCC. Firstly, 47 shared targets between HBV and HCC were screened as HBV-related HCC targets. Next, starting from 47 targets, the relevant chemical components and herbs were matched. A network containing 47 targets, 913 chemical components and 469 herbs was established. Then, the validated results showed that almost 80% of the herbs listed in chronic hepatitis B guidelines and primary liver cancer guidelines were included in the 469 herbs. Furthermore, functional analysis was conducted to understand the biological processes and pathways regulated by these 47 targets. The docking results indicated that the top 50 chemical components bound well to targets. Finally, the frequency statistical analysis results showed the 469 herbs against HBV-related HCC were mainly warm in property, bitter in taste, and distributed to the liver meridians. Taken together, a small library of 913 chemical components and 469 herbs against HBV-related HCC were obtained with a target-driven approach, thus paving the way for the development of therapeutic modalities to treat HBV-related HCC.

## Introduction

Hepatocellular carcinoma (HCC) is a serious health problem worldwide and the second most common cause of death from all malignancies (Bray et al., 2018). Hepatitis B virus (HBV) infection is still the most common etiological factor of HCC worldwide, especially in Asia (Catherine de Martel et al., 2015). Currently, essentially all HCC management guidelines recommend routine antiviral treatment to avoid HBV reactivation during treatment for HCC and reduce the recurrence of HCC after curative treatment (Sarin et al., 2016; European Association for the Study of the Liver, 2017; Terrault et al., 2018). However, a drug with repression effects on both HBV infection and HCC is not yet marketed. In addition, the current therapeutic regimens are far from optimal because of drug resistance, adverse, and toxic effects (Qiu et al., 2020). Novel multi-target medications with anti-HBV and anti-HCC activities are therefore urgently needed.

Traditional Chinese medicine (TCM) has always held a privileged position as an essential source of inspiration for discovering innovative drugs. Because they have multi-ingredient, multi-target properties that result in pharmacological synergism, it is possible that TCM had distinct advantages in dealing with multifactorial HBV infection and HCC (Kim and Kim, 2020; Zhang et al., 2020). In addition, TCM has time-honored theories about the diagnosis and treatment of liver diseases (Wang et al., 2012). In real clinical practice in China, TCM is part of the treatment regimen for HBV infection based on the syndrome of Chinese medicine. In hepatoma cancer therapy, TCM is mainly used to improve the anticancer drugs’ efficacy and reduce their toxicity.

Network pharmacology integrates system bioinformatics, multi-directional pharmacology and omics to develop new strategies and study drugs’ action mechanisms (Zhang et al., 2021). It can reveal the roles of pharmacological interventions, especially multi-target drugs. The overall concept of network pharmacology concurs with the “multi-ingredient and multi-target” theory of TCM. In general, based on the affirmative curative effects of TCM in treating a certain disease, network pharmacology was commonly used to predict the potential targets and underlying mechanisms of TCM interventions (Chen et al., 2021). In contrast, reverse network pharmacology is to use diverse public databases for initial disease target selection. Starting from the screened targets, effective medicine against this disease are subsequently explored (Dan et al., 2020; Dan et al., 2022). This specific target-driven assay allows us to identify medicine with a strong link to a disease. The strengths of target-driven approaches is that they are often simpler to execute than traditional phenotypic assays, operating with understanding of a drug’s specific biological hypothesis from an earlier stage, thus identifying more highly selective medicine (Croston, 2017). In addition, target-driven approaches are often higher in throughput than phenotypic assays, facilitating faster large-scale screening.

**Figure 1:**
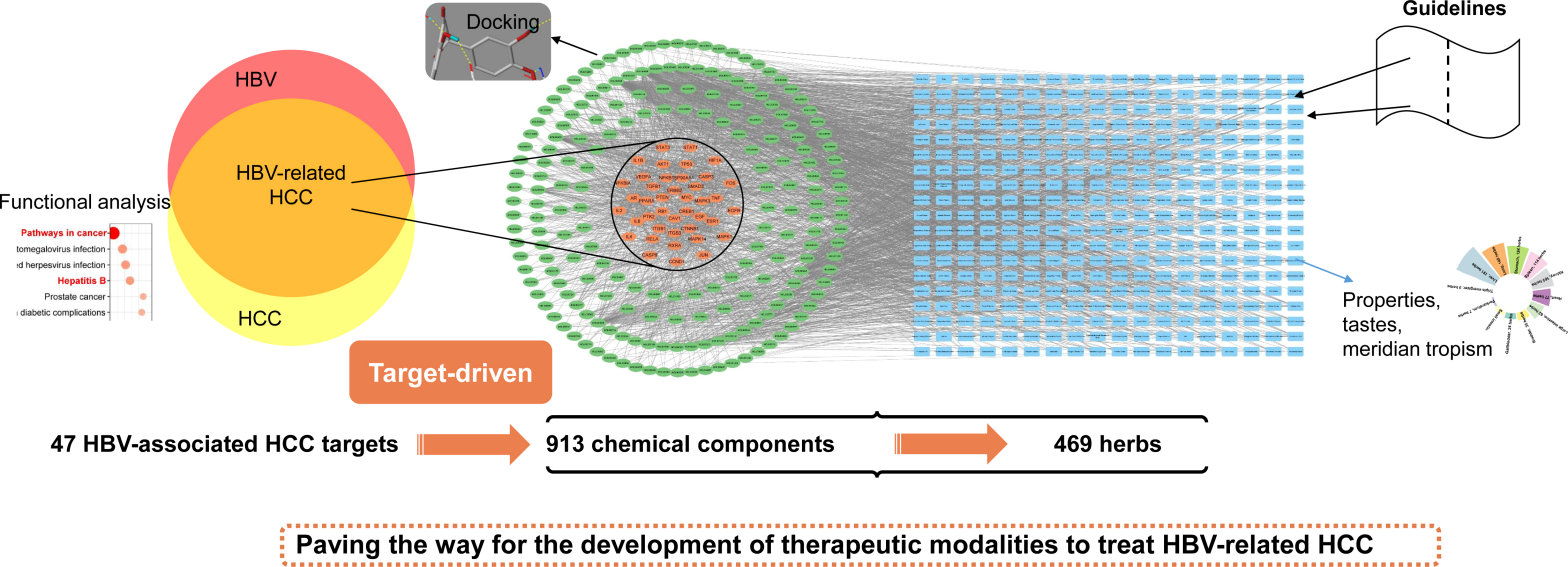
Workflow of this study.

The design of this study is shown in **Figure 1**. Firstly, the intersections of HBV and HCC targets were taken as HBV-related HCC targets. Next, starting from acquired targets, the corresponding chemical components and herbs were matched. A target-chemical component-herb network was established. The coverage of the herbs indicated in the guidelines was then used to evaluate the findings. Furthermore, functional analysis was performed for acquired HBV-related HCC targets. Molecular docking was applied to investigate the binding of obtained chemical components and targets. Finally, the frequency of herbs’ properties, tastes, and meridian tropisms was assessed. Consequently, the aim of this research was to apply a target-driven reverse network pharmacology strategy to gain systematic insight into the therapeutic potential of TCM against HBV-related HCC.

## Materials and methods

### Searching for HBV-related HCC targets

To obtain HBV targets and HCC targets, the Genecards database (Safran et al., 2010), and Therapeutic target database (Wang et al., 2020) were queried. The information on these targets was standardized using the Uniprot database (The UniProt Consortium, 2020). PPI data were obtained from the STRING database (Szklarczyk et al., 2021), with the term “Homo sapiens” restricted. A confidence level of 0.9 was selected. The CytoNCA plug-in of Cytoscape was applied to calculate the topological parameters (Shannon et al., 2003).

### Looking for relevant chemical components that act on HBV-related HCC targets

To find chemical components acting on relevant HBV-related HCC targets, the Traditional Chinese Medicine Systems Pharmacology Database and Analysis Platform (TCMSP) was searched (Ru et al., 2014). The screening conditions were as follows: oral bioavailability (OB) ≥ 30% and drug−likeness (DL) ≥ 0.18, half-life > 4 hours, molecular weight (MW) ≤ 500 Da, polar surface area (PSA) ≤ 140 Å2 and number of rotatable bonds (NBR) ≤ 10 (Lipinski et al., 2001; Huang et al., 2020). Given that the information provided by TCMSP was predicted by a computer, chemical components discarded in the initial screening were checked one by one to ensure that no relevant active chemical components were missed. These rechecked chemical components were selected as chemical components. The lists of the obtained chemical components and targets were introduced into Cytoscape to construct the target-chemical component network. A topological analysis was performed.

### Searching for herbs containing obtained chemical components

The TCMSP was searched to find herbs containing obtained chemical components. Herbs containing obtained chemical components were collected and an herb-chemical component network was constructed. Cytoscape software was utilized to construct the network of target-chemical component-herb.

### Enrichment analysis

Enriched gene ontology (GO) terms and the Kyoto Encyclopedia of Genes and Genomes (KEGG) pathway of 47 targets were carried out by Metascape (Zhou et al., 2019). P value < 0.01 was considered to indicate significant enrichment.

### Molecular docking

The crystal structures of the proteins were downloaded from the Protein Data Bank database (wwPDB Consortium, 2019). The 2D structures of chemical components were downloaded from the Pubchem database (https://pubchem.ncbi.nlm.nih.gov/). The PDB IDs for 46 targets are listed in **Table 1**. The molecular docking software Sybyl X-2.0 (Tripos, St. Louis, USA) was used. The Surflex-Dock program was used for the docking calculations.

**Table 1:** The PDB IDs for 46 targets.

Gene symbol	PDB ID	Gene symbol	PDB ID	Gene symbol	PDB ID
AR	4OHA	JUN	6Y3V	CTNNB1	3FQN
ESR1	7BAA	CASP8	4JJ7	ERBB2	5MY6
RXRA	6LB4	FOS	1S9K	NFKB1	7LFC
MAPK14	3LFF	IL2	5LQB	PPARA	6KAX
RELA	6NV2	HIF1A	4H6J	TGFB1	6OM2
CASP3	4QUJ	EGFR	5UG9	EGF	1NQL
TNF	5UUI	STAT3	6NJS	ITGB3	3T3P
TP53	3D06	STAT1	3wwt	SMAD3	5OD6
AKT1	4GV1	CREB1	5ZK1	CDKN1B	6ATH
IL6	1ALU	RB1	2R7G	CXCL12	4UAI
VEGFA	4GLS	MYC	6G6K	EP300	3BIY
IL1B	5R8Q	PTEN	7PC7	ITGB1	4WK0
MAPK1	4ZZN	HSP90AA1	5J2X	KRAS	6P0Z
IL4	4YDY	MAPK3	4QTB	RHOA	6V6U
NFKBIA	6Y1J	MAPK8	2XRW	–	–
CCND1	2W96	PTK2	6YOJ	–	–

### Herbal characteristics

A frequency analysis was done to draw the rules of herbs against HBV-related HCC. Various characteristics like channel tropism, flavor, and property were examined.

## Results

### Looking for and screening for HBV-related HCC targets

**Figure 2:**
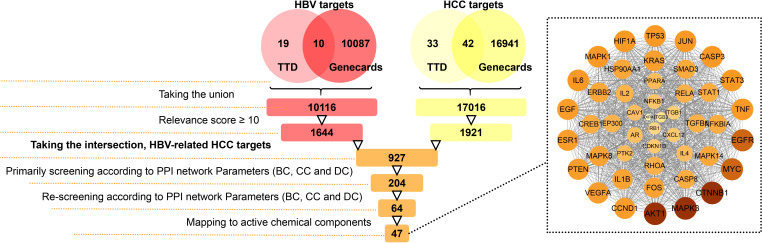
Procedure of searching and screening for HBV-associated HCC targets, which were derived by taking the intersection of HBV targets and HCC targets. Protein-protein interactions amongst the 47 targets were in the dashed box section.

An illustration of the screening process for HBV-related HCC targets is shown in **Figure 2**. Firstly, 10116 HBV target information was collected by taking the union of the results from TTD and GeneCards databases. 17016 HCC targets were obtained in a similar manner. Secondly, targets with a relevance score ≥ 10 were selected for research. There were still 1644 HBV targets and 1921 HCC targets. Thirdly, after taking the intersection of 1644 HBV targets and 1921 HCC targets, 927 HBV-related HCC targets were derived. The protein-protein interactions (PPI) network was an important tool for learning about cellular regulation and function (Valgardson et al., 2019). Fourthly, the PPI network of 927 targets was constructed by STRING and visualized by Cytoscape (Shannon et al., 2003). The topological parameters, including betweenness centrality (BC), closeness centrality (CC) and degree centrality (DC), were calculated for further screening. Only the nodes with higher values of DC (above twofold the median degree of all nodes), BC and CC (above the median value of all nodes) were identified as hub nodes which played a pivotal role within biological networks (Yu et al., 2018). After calculation, the thresholds for primarily screening were DC ≥ 22, BC ≥ 394, CC ≥ 0.041736 and 204 targets were derived. The thresholds for re-screening were DC ≥ 36, BC ≥ 2544, CC ≥ 0.04245, and this reduced the targets number to 64. Lastly, any targets that could not map to active chemical components on TCMSP and HIT website were discarded. Of these 64 targets, 47 were selected as HBV-related HCC targets (**Table 2**). The PPI network of these 47 HBV-related HCC targets are depicted in dashed boxed section in **Figure 2**.

**Table 2:** Forty-seven HBV-associated HCC targets ^a^.

Gene symbol	Uniprot ID	Protein name
AR	P10275	Androgen receptor
ESR1	P03372	Estrogen receptor
RXRA	P19793	Retinoic acid receptor RXR-alpha
MAPK14	Q16539	Mitogen-activated protein kinase 14
RELA	Q04206	Transcription factor p65
CASP3	P42574	Caspase-3
TNF	P01375	Tumor necrosis factor
TP53	P04637	Cellular tumor antigen p53
AKT1	P31749	RAC-alpha serine/threonine-protein kinase
IL6	P05231	Interleukin-6
VEGFA	P15692	Vascular endothelial growth factor A
IL1B	P01584	Interleukin-1 beta
MAPK1	P28482	Mitogen-activated protein kinase 1
IL4	P05112	Interleukin-4
NFKBIA	P25963	NF-kappa-B inhibitor alpha
CCND1	P24385	G1/S-specific cyclin-D1
JUN	P05412	Transcription factor AP-1
CASP8	Q14790	Caspase-8
FOS	P01100	Proto-oncogene c-Fos
IL2	P60568	Interleukin-2
HIF1A	Q16665	Hypoxia-inducible factor 1-alpha
EGFR	P00533	Epidermal growth factor receptor
STAT3	P40763	Signal transducer and activator of transcription 3
STAT1	P42224	Signal transducer and activator of transcription 1-alpha/beta
CREB1	P16220	Cyclic AMP-responsive element-binding protein 1
RB1	P06400	Retinoblastoma-associated protein
MYC	P01106	Myc proto-oncogene protein
PTEN	P60484	Phosphatidylinositol 3
CAV1	Q03135	Caveolin-1
HSP90AA1	P07900	Heat shock protein HSP 90-alpha
MAPK3	P27361	Mitogen-activated protein kinase 3
MAPK8	P45983	Mitogen-activated protein kinase 8
PTK2	Q05397	Focal adhesion kinase 1
CTNNB1	P35222	Catenin beta-1
ERBB2	P04626	Receptor tyrosine-protein kinase erbB-2
NFKB1	P19838	Nuclear factor NF-kappa-B p105 subunit
PPARA	Q07869	Peroxisome proliferator-activated receptor alpha
TGFB1	P01137	Transforming growth factor beta-1 proprotein
EGF	P01133	Pro-epidermal growth factor
ITGB3	P05106	Integrin beta-3
SMAD3	P84022	Mothers against decapentaplegic homolog 3
CDKN1B	P46527	Cyclin-dependent kinase inhibitor 1B
CXCL12	P48061	Stromal cell-derived factor 1
EP300	Q09472	Histone acetyltransferase p300
ITGB1	P05556	Integrin beta-1
KRAS	P01116	GTPase KRas
RHOA	P61586	Transforming protein RhoA

a The targets are sorted in decreasing order of degree value.

### Screening for relevant chemical components and constructing the target-chemical component network

**Figure 3:**
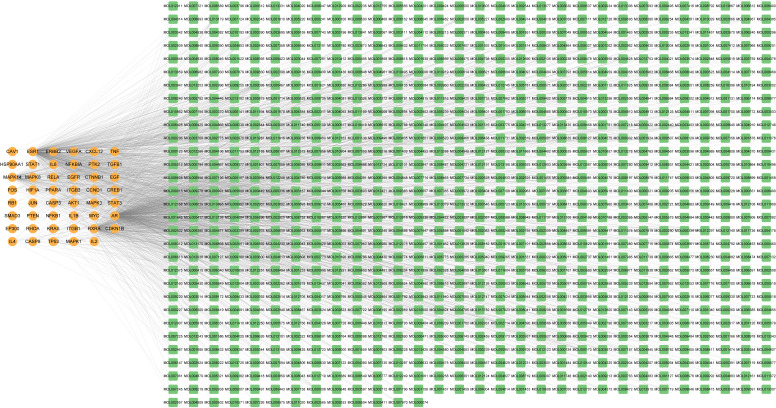
The 47 HBV-associated HCC targets-913 chemical components network. Orange nodes represented the targets, while green nodes represented chemical components. The edges indicated the interaction between targets and chemical components.

Forty-seven HBV-related HCC targets were mapped to 2013 chemical components. To make the present study more close to the real world, chemical components which met ADME and Lipinskir rules criteria (OB ≥ 30%, DL ≥ 0.18) were left. Other research-worthy chemical components were derived based on the official “Chinese Pharmacopoeia” (2020 edition). Eventually, 942 chemical components were identified. Later, a target-chemical component network was developed (**Figure 3**). The target-chemical component network included 960 nodes and 1854 edges (Zhu et al., 2019). Orange nodes represented the targets, while green nodes represented chemical components. The edges indicated the interaction between targets and chemical components. The larger the node size, the greater the degree of connectivity.

### Screening for relevant herbs and constructing the target-chemical component-herb network

**Figure 4:**
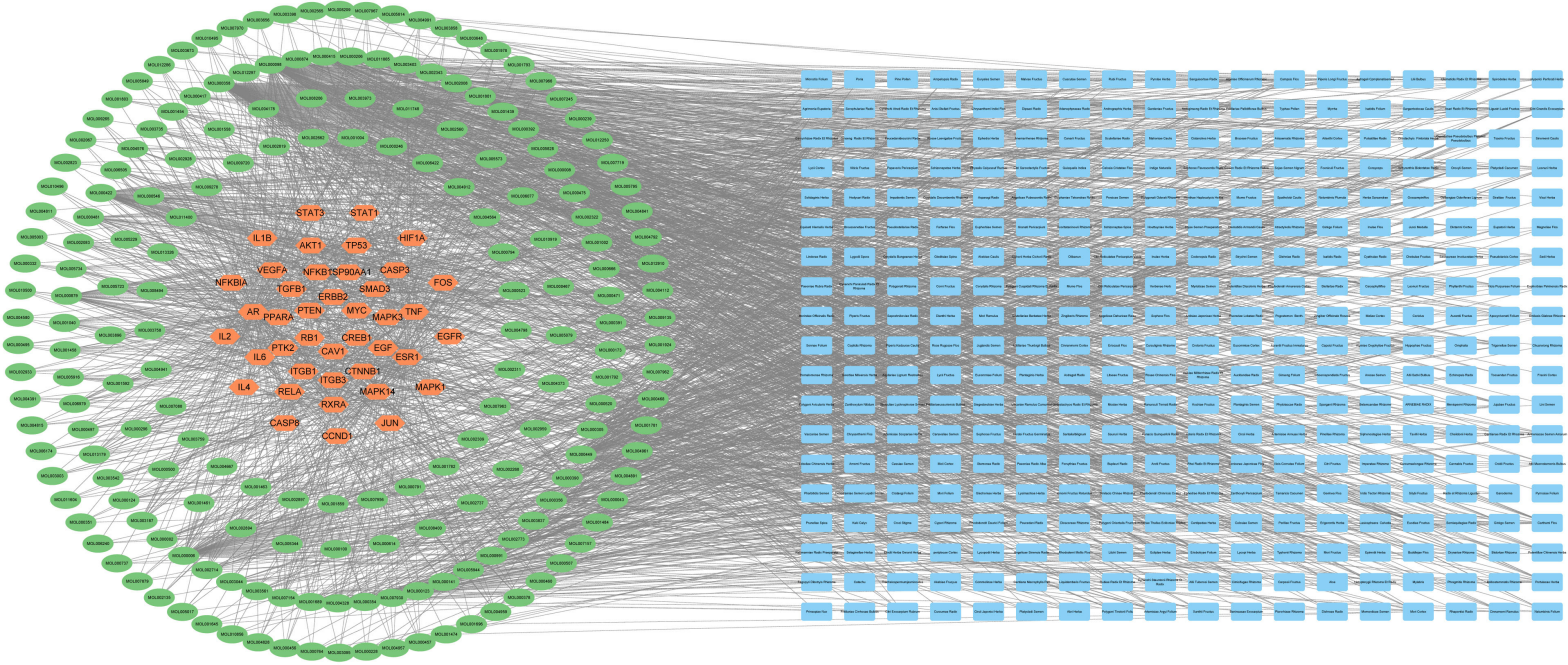
The 47 targets-913 chemical components-469 herbs network. Orange, green, and blue nodes represented the targets, chemical components, and herbs, respectively.

We not only focus on the chemical components, but also combined with the corresponding herbs. Among the above-identified 942 chemical components (components in herbs), no corresponding herbs for 29 chemical components could be found. The remained 913 chemical components were provided in **Supplementary Material** and mapped to 469 herbs. A full list of 469 herbs was available from the **Supplementary Material**. That is to say, 469 herbs were associated with 47 targets by linking 913 chemical components. The network of 47 targets-913 chemical components-469 herbs was established by combining target-chemical component network and herb-chemical component network (**Figure 4**). To make the network more concise and more intuitive, chemical components and herbs with degree values less than six were hidden. The target-chemical component-herb network consisted of 1345 nodes and 4619 edges. Like **Figure 3**, orange and green nodes represented targets and chemical components. Blue nodes in **Figure 4** represented mapped 469 herbs.

To assess the reliability of the final results, the recommended herbs in the guidelines for diagnosis and treatment of primary liver cancer in China (2020 Edition) and the clinical guidelines of diagnosis and treatment of chronic hepatitis B with traditional Chinese medicine (2018 Edition) were summarized. After removing duplicates, there were 112 herbs listed in these two guidelines. After comparison, 86 out of 112 herbs (almost 80%) were included in the 469 herbs, suggesting that the herbs that resulted from target-driven reverse network pharmacology were not divorced from clinical practice. Through design, new herbal formulae against HBV-related HCC could be developed based on the individual synergistic nature of each herb and the “Jun-Chen-Zuo-Shi” (also known as “sovereign-minister-assistant-courier”) rule.

### The mechanisms of the 47 targets

**Figure 5:**
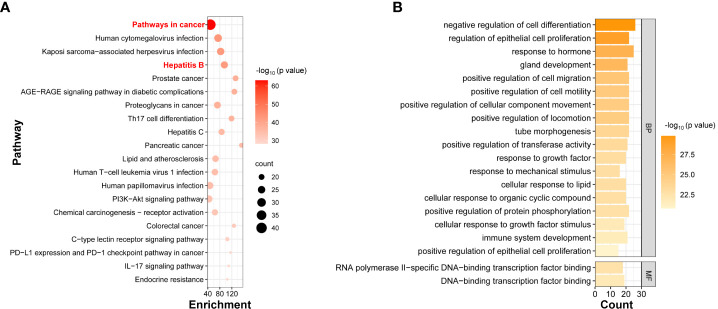
The functional enrichment analysis of 47 targets. **(A)** The top 20 KEGG pathways by p value. The bubble size indicated the number of targets clustered, and the color indicated the p value of the enrichment analysis. **(B)** The GO terms were sorted according to p value, with the most significantly enriched terms at the top. BP, biological processes. MF, molecular functions. The length of each bar indicated the number of targets clustered, and the color indicated the p value of the enrichment analysis.

To determine the potential mechanisms that the 47 HBV-related HCC targets were involved in, functional enrichment analysis was conducted. Pathway enrichment from the KEGG database showed that 174 pathways passed the filtering criteria, and the top 20 pathways according to p value were in **Figure 5A**. The most significantly enriched pathway was “pathways in cancer” with 40 targets clustered. This pathway was reported playing important tasks in HCC progression (Zhou et al., 2015). Three pathways including “hepatitis B” pathway were selected as the second group of most-significantly enriched pathways with quite similar p values and gene numbers. KEGG analyses highlighted the 47 targets that were not only topological connectors but also functional connectors in the crosstalk network of HBV and HCC. In addition, “human cytomegalovirus infection” and “kaposi sarcoma-associated herpesvirus infection” were also enriched in the second group. The human cytomegalovirus was a DNA virus that belonged to the herpes virus family. Therefore, the application of the matched chemical components and herbs based on these 47 targets may not be limited to HBV disease but also extend to human cytomegalovirus and kaposi sarcoma-associated herpesvirus infection.

The 47 targets were assigned to 1416 Gene Ontology (GO) terms, including 1280 biological process terms, 77 molecular function terms, and 59 cellular component terms. The top 20 GO terms ranked by p value are in **Figure 5B**. The types of the top 20 GO terms were biological processes and molecular functions instead of cellular components. The most significantly enriched terms were closely related to cell differentiation, proliferation, and migration processes, which were strongly associated with cancer occurrence, development, and metastasis (Sack et al., 2018). These enriched terms were also observed in previous HBV-related HCC literature (Wang et al., 2017; Fan et al., 2017; Zhang et al., 2021). Two molecular function terms most significantly enriched were associated with DNA-binding transcription factor binding, which was also closely linked to cancer (Bai et al., 2020).

### Binding affinities between top 50 chemical components and targets

**Figure 6:**
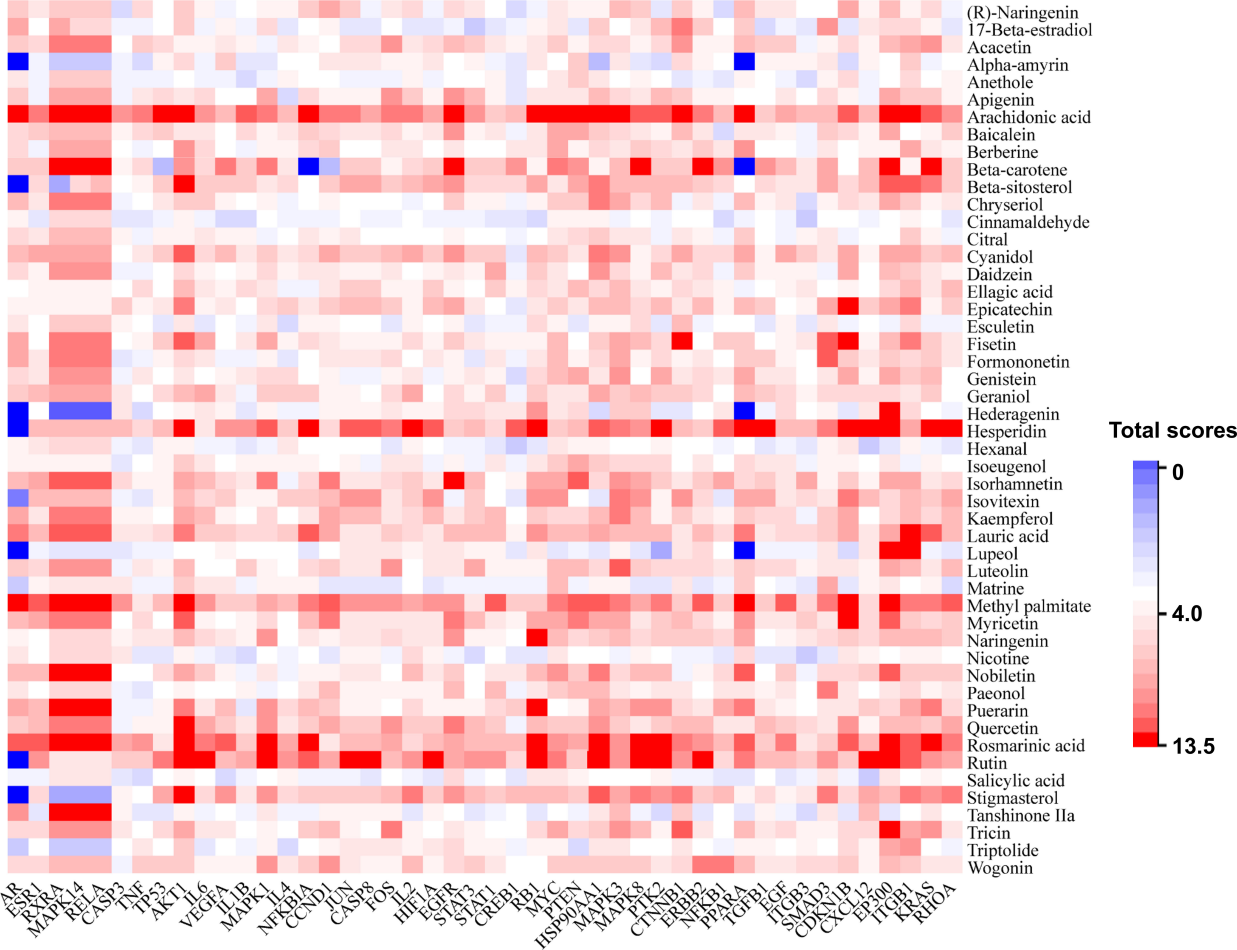
The binding scores between 46 targets (x axis) and the top 50 chemical components ranked by node degree value (y axis). Colors indicated different scores. (red, higher than 4.0; blue, less than 4.0).

Molecular docking can foresee binding modes of small molecules with target proteins and predict molecular interactions (Saikia and Bordoloi, 2019). According to the rule of Sybyl, the total score shows the binding affinity between the small molecule and a potential target. A higher total score suggests a closer interaction between small molecule and potential target (Li et al., 2022; Zhang et al., 2019). A total score ≥ 4.0 indicates that the small molecule binds well to the targets (Guo et al., 2022). Considering that 3D structure for CAV1 was not available in the PDB database, docking studies were performed between 46 targets and the top 50 chemical components ranked by node degree value. As shown in **Figure 6**, 2300 scores were obtained, and the obtained total scores were presented on a color scale (red, higher than 4.0; blue, less than 4.0). The average score of 2300 sets of receptor-ligand docking results was 5.8, indicating good binding affinities between these 50 chemical components and the 46 targets in the target-chemical component network.

**Figure 7:**
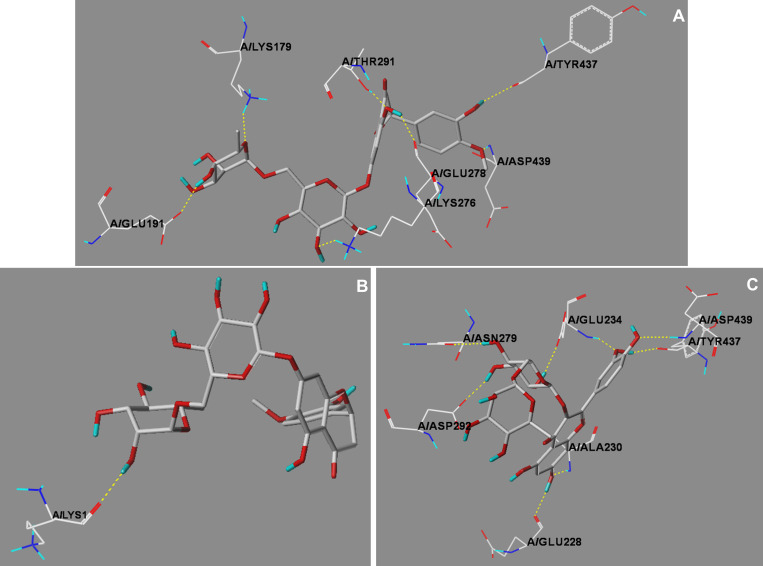
The hydrogen bonding plots of **(A)** AKT1-hesperidin, **(B)** CXCL12-hesperidi, and **(C)** AKT1-rutin. All ligands were depicted in a capped stick representation, while the interacting residues were shown as lines. The hydrogen bonds were yellow dashed lines.

The top three binding score values were yielded by the binding of AKT1-hesperidin (score: 13.6), CXCL12-hesperidin (score: 12.4) and AKT1-rutin (score: 11.9). The corresponding hydrogen bonding plots are shown in **Figure 7**. It can be seen from **Figure 7** that each ligand (chemical component) interacted with multiple residues of the target through at least one hydrogen bond. On the other hand, all these three combinations were not discovered in the target-chemical component network. That is, the potential chemical component-target combinations may be far more diverse than that included in the TCMSP database. There were still large amounts of interactions between active TCM chemical components and HBV-related HCC targets waiting to be further mined. The docking results could provide rapid and inexpensive technique support for future laboratory screening of related chemical component and herbs.

### Properties, tastes, and meridian tropisms of herbs against HBV-related HCC

**Figure 8:**
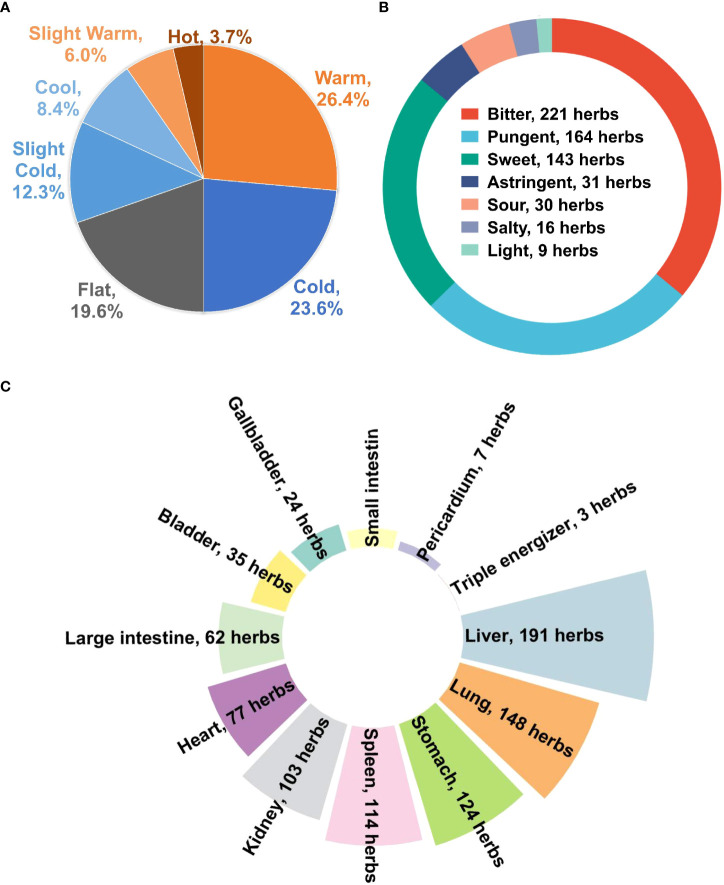
**(A)** Properties, **(B)** tastes, and **(C)** meridian tropism of herbs against HBV-associated HCC.

**Figure 8** represented the frequency analysis results, which summarized the rules of 381 herbs that could be found in the Chinese Pharmacopoeia (2020 edition). In TCM, the properties of herbs mean their effects, which are classified into cold, cool, even, hot, and warm. The properties of Chinese medicinal herbs served as the foundation for herb analysis and clinical application (Huang et al., 2018). As shown in **Figure 8A**, the properties of 381 herbs were mainly warm (26.4%), followed by cold (23.6%). The total proportion of these two types of medicines accounted for more than 50% of all herbs. According to TCM theory, warm medicine can be warming and nourishing, thus reinforcing healthy Qi and helping to eliminate pathogenic factors (Liu et al., 2019). Cold medicine generally has a clearing action to get rid of the body of excess substances to regulate the balance of the body (Xia et al., 2020).

Herbs are also categorized according to their flavors, including bitter, pungent, salty, sour, and sweet. In terms of tastes, bitter accounted for the largest number (**Figure 8B**). The second was pungent. Bitter medicines have downward effects such as clearing away dampness and purging, whereas pungent medicines have outward and upward effects of dispersing (Xia et al., 2020). Using bitter medicines and pungent medicines in combination can dissolve stagnation of blood. This makes blood circulate smoothly, which can relieve pain.

Meridian tropisms are TCM organ systems. Namely, the target organs of herbs, like the heart, spleen, and liver (Xu et al., 2019). The theory of meridian tropism plays an important role in the clinical selection of TCM. Concerning meridian tropisms, half of the herbs were liver meridian, which matched the disease region of HBV and HCC (**Figure 8C**). The second most matched meridian was the lung meridian. The liver governs regulating and ascending. The lung governs purification and descending. Both organs complement each other to harmonize qi, blood, and body fluid and to restore the non-pathological state (He et al., 2019).

## Discussion

The diverse and growing knowledge of genomic information, multilevel biological interactions, and disease mechanisms facilitates the elucidation and discovery of new potential targets at which novel treatment development could be directed (Hoehe and Morris-Rosendahl, 2018). Target-driven reverse network pharmacology is one such strategy to use geneomewide target-ligand interaction networks in TCM to link genetic and drug data together (Dan et al., 2020).

Before the introduction of target-driven approaches, drug discovery was based primarily on phenotypic assays, often with limited information about the molecular mechanisms of disease. It is often necessary to characterize the mechanisms of active molecules identified in phenotypic screens to assist with the optimization of a candidate. Moreover, phenotypic assays exhibited lower throughput than target-driven approaches. In contrast, target-driven approaches allow significantly faster drug discovery and development than conventional phenotypic approaches.

Docking results suggested that the three strongest binding were AKT1-hesperidin, CXCL12-hesperidin and AKT1-rutin. Interestingly, these three sets of target-chemical component interaction were in line with previous experimental data. Hesperidin is a kind of citrus flavonoids and numerous studies have delineated anti-HBV and anti-HCC activities of hesperidin (Li et al., 2018; Parvez et al., 2019; Khuanphram et al., 2021). It has been demonstrated that AKT1 phosphorylation in RBL-2H3 cells and male rats can be suppressed by hesperidin (Kobayashi and Tanabe, 2006; Li et al., 2018). Hesperidin has been also reported attenuating the secretion of CXCL12 from A549 cells in a dose-dependent manner by ELISA method (Xia et al., 2018). Rutin, a flavonoid widely found in plants, exhibits anti-HCC activities in Wistar rats (Pandey et al., 2021). Rutin can regulate phosphorylated AKT1 expression in different tissues (Li et al., 2022; Liang et al., 2018; Fei et al., 2019). With the advent of increasing large-scale data acquisition, the application of network pharmacology in herbal formulas has provided a new horizon in the study of related domains such as active compound discovery, mechanism research, quality control, and others. Network pharmacology is expected to help traditional herbal medicine transition from experience-based medicine to evidence-based medicine (Li et al., 2022). However, it has inherent limitations, such as a lack of clinical data (Vogt and Mestres, 2019; Wu et al., 2021). In this respect, the major limitation of this work is a lack of *in vivo* and *in vitro* experiments. Experimental validations are needed to further verify our findings in later studies.

## Conclusion

To our knowledge, this was the first attempt to systematically study HBV-related HCC treatment with TCM using target-driven reverse network pharmacology. A small library of 913 chemical components and 469 herbs against HBV-related HCC were acquired, with the hope of providing theoretical reference for more therapeutic options and may eventually benefiting clinical practice. Moreover, our studies promise to greatly expand the previous understanding of combined use of TCM-derived and western medicine.

## Data availability statement

The original contributions presented in the study are included in the article/**Supplementary Material**. Further inquiries can be directed to the corresponding authors.

## Author contributions

Conceptualization and writing, XY and YQ. Software, ZH, JL, and RD. Validation, YQ. All authors contributed to the article and approved the submitted version.

## Funding

This work was supported by Shanxi Research Program of Application Foundation [No. 20210302123266]; Shanxi Medical University Second Hospital Doctoral Sustentation Fund [No. 201501-4].

## Conflict of interest

The authors declare that the research was conducted in the absence of any commercial or financial relationships that could be construed as a potential conflict of interest.

## Publisher’s note

All claims expressed in this article are solely those of the authors and do not necessarily represent those of their affiliated organizations, or those of the publisher, the editors and the reviewers. Any product that may be evaluated in this article, or claim that may be made by its manufacturer, is not guaranteed or endorsed by the publisher.
